# Single-step fabrication of quantum funnels via centrifugal colloidal casting of nanoparticle films

**DOI:** 10.1038/ncomms8772

**Published:** 2015-07-13

**Authors:** Jin Young Kim, Valerio Adinolfi, Brandon R. Sutherland, Oleksandr Voznyy, S. Joon Kwon, Tae Wu Kim, Jeongho Kim, Hyotcherl Ihee, Kyle Kemp, Michael Adachi, Mingjian Yuan, Illan Kramer, David Zhitomirsky, Sjoerd Hoogland, Edward H. Sargent

**Affiliations:** 1 Department of Electrical and Computer Engineering, University of Toronto, 10 King's College Road, Toronto, Ontario M5S 3G4, Canada; 2 Fuel Cell Research Center, Korea Institute of Science and Technology, Seoul 136-791, Republic of Korea; 3 Nanophotonics Research Center, Korea Institute of Science and Technology, Seoul 136-791, Republic of Korea; 4 Center for Nanomaterials and Chemical Reactions, Institute for Basic Science, Daejeon 305-701, Republic of Korea; 5 Department of Chemistry, Korea Advanced Institute of Science and Technology, Daejeon 305-701, Republic of Korea; 6 Department of Chemistry, Inha University, Incheon 402-751, Republic of Korea

## Abstract

Centrifugal casting of composites and ceramics has been widely employed to improve the mechanical and thermal properties of functional materials. This powerful method has yet to be deployed in the context of nanoparticles—yet size–effect tuning of quantum dots is among their most distinctive and application-relevant features. Here we report the first gradient nanoparticle films to be constructed in a single step. By creating a stable colloid of nanoparticles that are capped with electronic-conduction-compatible ligands we were able to leverage centrifugal casting for thin-films devices. This new method, termed centrifugal colloidal casting, is demonstrated to form films in a bandgap-ordered manner with efficient carrier funnelling towards the lowest energy layer. We constructed the first quantum-gradient photodiode to be formed in a single deposition step and, as a result of the gradient-enhanced electric field, experimentally measured the highest normalized detectivity of any colloidal quantum dot photodetector.

## Abstract (web summary)

Centrifugal casting is widely used to fabricate functional materials that exhibit built-in spatial gradients due to the variation in material density. Kim *et al.* use this method to prepare colloidal quantum dot films for the first time, based on which photodetectors with gradient bandgap are built.

## Graphical abstract

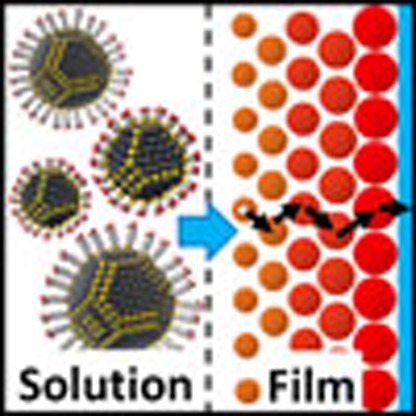

Centrifugal casting^1^,^2^,^3^,^4^,^5^,^6^ exploits centrifugal acceleration to distribute and ultimately cast material in a mould of widely selectable area and thickness. This manufacturing technique has been exploited when large size, low cost and extremely high materials utilization are needed. Metals, ceramics and polymers can be cast with this process, and novel composites can be formed by introducing additional compounds.

Since centrifugal forces act continuously during material casting, solutions containing mixtures of constituent materials will see the various components deposited at different velocities, as determined by the materials' densities^7^,^8^,^9^. In most prior applications, the resultant gradient films represent a disadvantage of centrifugal casting, since many applications prize homogeneity rather than segregation^10^,^11^.

We take the view that materials and devices that benefit from spatial gradients would represent attractive systems in which to feature the excellent scalability and materials utilization efficiency of the process. Until the present work, functional gradient materials made via the centrifugal casting process have focused on bulk metallic, ceramic composite structures^12^,^13^,^14^,^15^,^16^,^17^.

We instead deploy centrifugal casting to form controllably spatially graded architectures that feature nanoscale-tuned particles and properties. We focus on quantum dots, nanoparticles whose diameter programs their HOMO and LUMO energy levels relevant to optoelectronic behaviour^18^,^19^,^20^,^21^,^22^,^23^. Synthetic advances in nanoparticles such as a narrow-size distribution of better than 5% and precise control of size tunability have allowed size tuning to become a powerful lever to control function^24^,^25^.

We focus in particular on building functional-graded materials based on colloidal quantum dots (CQDs). The resultant CQD solids feature nanoparticles whose diameters change programmably over the vertical extent of the film. The resultant device allows us to engineer the electronic transport of charge carriers (for example, electrons) and focus their egress in the direction of the desired electron-accepting material^26^,^27^.

## Results

### Fabrication of CQD film via C3 processing

A first necessary step towards the goal of centrifugal colloidal casting (C3) was to create a stable colloid that, once directly deposited, would produce a high-performance optoelectronic material. We developed a method to prepare a stable solution of CQDs capped using a wide range of short ligand molecules.

**Figure 1:**
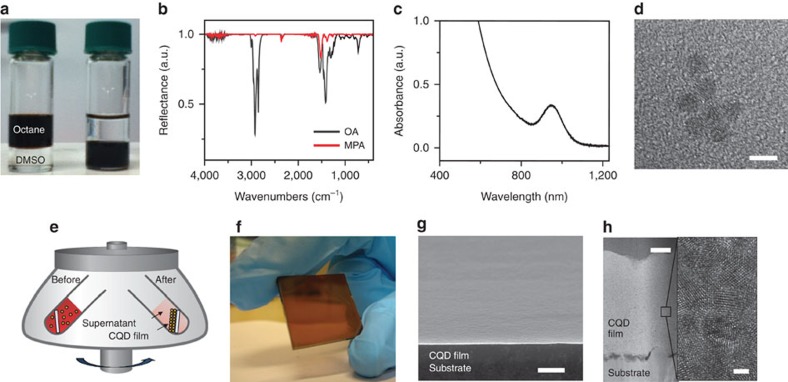
Direct formation of CQD solids via centrifugal casting of CQD ink. (**a**) Photograph showing the ligand exchange process of OA–PbS CQD solution with MPA molecules. Red coloured colloidal dispersion of OA–PbS CQDs undergoes the phase transfer from octane (left) to DMSO (right) on exchange of the original organic surface ligands with MPA. (**b**) Fourier transform infrared spectra of PbS CQDs capped with the original OA ligands (black), and with MPA ligands (red). (**c**) Absorption spectrum of 3.3 nm PbS CQDs capped with MPA ligands dispersed in DMSO. (**d**) TEM image of 3.3 nm PbS CQDs capped with MPA ligands. Scale bar, 5 nm. (**e**) Schematic diagram showing the centrifugal casting method to fabricate CQD films. Before centrifugation, substrate is inserted and fixed in a centrifuge tube held at ∼30° containing suspensions of CQDs in DMSO. During the centrifugal rotation, particles in the suspension start to settle out of the fluid in which they are entrained, and assemble on the substrate. After complete sedimentation of particles, the supernatant liquid is carefully decanted from the tube without disturbing the CQD precipitate. (**f**) Photograph of MPA-exchanged PbS CQD film via centrifugal casting method. (**g**) Scanning electron microscopy (SEM) shows that films are crack-free and macroscopically uniform over large areas. Scale bar, 500 nm. (**h**) A cross-sectional TEM image of a MPA-exchanged 4 nm PbS CQD film sample prepared by focused ion beam milling (left). Scale bar, 100 nm. Magnified TEM image of the MPA–PbS CQDs film (right). Scale bar, 2 nm.

We illustrate the method using the specific example of mercaptopropionic acid (MPA), a molecule of particular interest since it led to the best performance achieved in this work. We devised a two-phase exchange to replace long and bulky ligand capped CQDs in a non-polar solvent to a short linker-capped system in a polar solvent^28^,^29^,^30^. We combined solutions of as-synthesized oleic acid (OA)-capped PbS CQDs in a non-polar solvent (for example, octane or toluene) with a solution of short MPA ligands in a polar solvent (for example, dimethylsulfoxide (DMSO) or dimethylformamide). After mixing the two-phase mixture containing immiscible layers of polar and non-polar solvent, we observed complete transfer of CQDs from the non-polar to the polar solvent (Fig. 1a). The original organic ligands remain in the non-polar phase. The ligand exchange is complete, confirmed by comparing the infrared absorption spectra for CQDs before and after the ligand exchange procedure (Fig. 1b). After exchange, C–H stretching modes distinctive to OA are no longer present in the spectra. The excitonic features of the PbS CQDs in the optical absorption spectra are fully preserved following the ligand exchange (Fig. 1c). No changes in CQD size and shape were seen, but dense packing was observed, again consistent with complete OA removal (Fig. 1d, Supplementary Fig. 1).

The product of this colloidal manipulation was a nanoparticle dispersion ready for direct deposition and use as an active optoelectronic material. To implement C3, we placed a substrate in a centrifuge tube, filled the tube with CQD ink (Fig. 1e) and optimized the choice of tilt angle (∼30°) and rotation speed (∼15,000 *g*) to achieve uniform casting. We found that we could control the centrifugal sedimentation rate of CQDs systematically through the careful addition of small amounts of marginal solvents into the CQD ink. The centrifugation time was determined empirically by observing when the CQD ink became colourless. This supernatant was then discarded and the substrate removed from the tube and further dried at room temperature in air. Given the resultant complete materials utilization, the film thickness was systematically controlled by varying the total volume of CQDs in the solution.

C3 enabled the facile deposition of macroscopically uniform CQD films directly from solution (Fig. 1f, Supplementary Fig. 2). The method led to compact, void- and crack-free CQD films (Fig. 1g). CQD films built up using the centrifugal method did not contain the segregated interfaces characteristic of layer-by-layer deposited films, but instead were homogeneous when visualized using transmission electron microscopy (TEM; Fig. 1h). Grazing-incidence small-angle X-ray scattering (GISAXS) showed no scattering peak in the CQD films, suggesting the absence of in-plane and out-of-plane ordering (Supplementary Fig. 3). The absorption spectra for the films of MPA-exchanged PbS CQDs closely resembled spectra of the colloidal solution, indicating that the quantum-confined bandgap was fully retained and that the CQDs did not aggregate during the casting process (see Supplementary Fig. 4). In films, the ∼30 meV redshift compared with in solution points to the partial relaxation of the quantum confinement due to strong electronic coupling among the particles, combined with the effect of an increased dielectric constant^31^. The magnitude of the absorption coefficient in the centrifuged film compared favourably with that of the film prepared with a layer-by-layer method, indicating dense packing of CQDs inside the film.

To the best our knowledge, this is the first experimental demonstration of single-step direct fabrication of CQD films employing a stable CQD ink whose constituent nanoparticles were coated using electron-transport-favouring short ligand molecules. We were able to fabricate from a wide range of CQD solutions on a range of substrates, a finding that suggests broad application of C3 processing (see Supplementary Fig. 5).

### CQD size characteristics in C3-created films

Our development of C3 was motivated by the desire to impart gradual changes in nanoparticle diameter, and thus bandgap, via the action of the centrifugal force. We hypothesized that assembling CQDs under an applied centrifugal force would stratify the film by nanoparticle size, giving rise to an additional internal electric field induced by the graded topology that would promote carrier transport.

**Figure 2:**
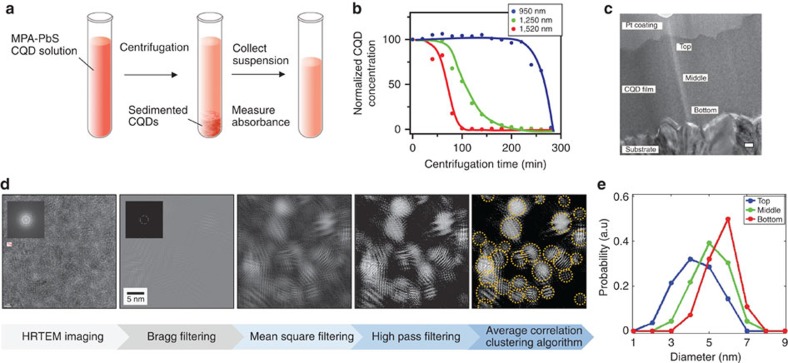
Experimental analysis of grading in CQD solids formed by C3. (**a**) Schematic of experiment used to measure sedimentation rates of CQD particles in solution during centrifugation. The solution was used with a mixture of three different-sized MPA–CQDs with excitonic peaks at 950, 1,250 and 1,520 nm. (**b**) Sedimentation rates of the particles were determined by measuring the change in the particle concentrations in the suspension from the absorbance. Absorption measurements recorded during centrifugation showed that particles of different sizes sediment at different rates, with the larger particles precipitating faster than smaller particles. Lines are added as a guideline for visualization. (**c**) A cross-sectional TEM image of the C3-created CQD film fabricated with the mixed solution of three different-sized MPA-CQDs with excitonic peaks of 950, 1,250 and 1,520 nm. The film was coated with Pt layer to avoid charging. (**d**) Image analysis algorithm for the quantitative recognition of the particle size in the high-resolution TEM images of MPA–PbS CQD structures (refer to Supplementary Note 1 for details). (**e**) Spatial size distribution of the MPA–PbS particle sizes in the film for locations from the top, middle and bottom of films.

We therefore studied the conditions for spatial CQD gradient formation, first examining the sedimentation rate of differently sized particles in a mixture of CQDs (Fig. 2a). We obtained absorption spectra of the colloidal dispersion at many stages along the centrifugal deposition process. The studied solution contained a mixture of three different-sized MPA–CQDs with excitonic peaks of 950, 1,250 and 1,520 nm, making clear comparisons possible. The individual particle-based transitions in the experiment were resolved in the absorption spectra, with deconvolution carried out taking into account the superposition of partially overlapping spectra (see Supplementary Fig. 6). The relative distribution of each CQD type within the ensemble as a function of centrifugation time is presented in Fig. 2b. CQDs of different sizes in the dispersion sediment at different rates, with the larger and heavier particles sedimenting faster than the smaller ones.

We then turned to examine the spatial distribution of particle sizes throughout the thickness of the film. We obtained cross-sectional TEM images for locations from the top, middle and bottom of films (Fig. 2c). We developed and applied an image analysis algorithm based on Fourier filtering to quantitate the size of nanoparticles seen in high-resolution TEM images (Fig. 2d; Supplementary Figs 7–9; Supplementary Table 1; Supplementary Note 1). The imaging processing technique moves a small mask along a circular path in the Fourier spectrum of a TEM image and computes, at each angular step, an inverse Fourier spectrum. The procedure extracts the amplitude from the Fourier reconstructions and regenerates a sum picture that is a real-space map of the local amplitude. The numerical routine has the benefit that it calculates separately the amplitude and phase of the lattice fringes of the CQDs as a function of position. The information is robust with respect to noise produced by the presence of amorphous regions such as ligand molecules and free space. The images so produced are in a form suitable for statistical analysis, and the method has been demonstrated in discriminating the size and shape distribution of nanocrystalline materials^32^,^33^,^34^.

The results confirm that top of the film is comprised of particles having average diameter 4 nm, and the bottom of the film of nanoparticles having average diameter 6 nm (Fig. 2e).

### Carrier funnelling in C3-created film

We then sought to build gradient carrier funnelling films and characterize the direction of charge transport. For PbS CQDs, even though electron- and hole-effective masses are similar, band-edge energies are reported to undergo asymmetric change with varying particle size. Empirical data confirm that valence band is almost unchanged and the vast preponderance of confinement energy goes to conduction band^35^. Funnelling in the graded PbS CQD structures is therefore expected to occur predominantly in the conduction band.

**Figure 3:**
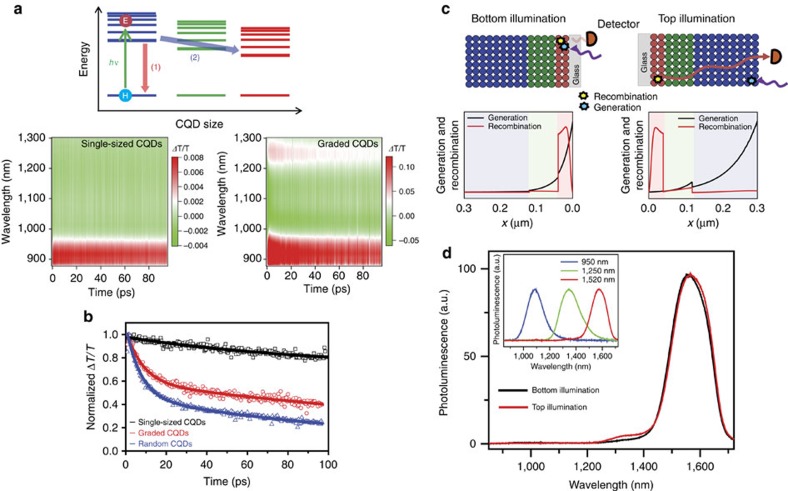
Funnel characteristics in graded CQD solids formed by C3. (**a**,**b**) Exciton recombination and charge transfer in graded CQD solids (**a**, top). In single-sized CQDs, photogenerated electrons undergo ultrafast intraband relaxation and subsequent exciton recombination. In contrast, both (1) exciton recombination and (2) charge transfer to a layer of larger CQDs can occur in graded CQDs. Time evolution of transient absorption (TA) spectra for single-sized CQDs (left) and graded CQDs (right) (**a**, bottom). The positive signal at wavelengths smaller than 950 nm is observed for both single-sized CQDs and graded CQDs and arises from the bleaching of the lowest exciton transition in the smallest CQDs. (**b**) Time profiles of transient absorption signals at 950 nm probe wavelength (points) and their exponential fits (lines) for single-sized (black), graded (red) and randomly mixed (blue) CQDs. (**c**,**d**) Photoluminescence studies of the graded CQD film formed on glass substrate under illumination from the small-bandgap side (bottom illumination) and the large-bandgap side (top illumination) of the graded film (**c**, top). For the measurements, films were illuminated with a laser beam at *λ*=440 nm, and the emission was collected from the same side where light is illuminated. The CQD layer was composed of ∼180-, ∼80- and ∼40-nm thick CQD films with corresponding excitonic peaks at 950 (blue), 1,250 (green) and 1,520 nm (red), respectively. (**d**) The photoluminescence spectra when illuminating from either side overlap closely. The inset shows the photoluminescence spectral profile emitted from each CQD materials. From this we conclude that photogenerated charge carriers are efficiently funnelled to the small-bandgap material in the graded film. This is consistent with generation and recombination plots of both structures predicted using the electronic materials parameters associated with the CQD films (**c**, bottom). Colour-shaded regions are representative of film thicknesses for each size of CQD.

To examine the characteristics of carrier funnelling in C3-created films, we carried out a femtosecond transient absorption spectroscopy experiment on CQD solids. When CQDs are excited via the absorption of light from a visible laser pulse of which the photon energy exceeds the bandgap energy, an electron and a hole are generated. In single-sized CQDs, the photogenerated electron undergoes ultrafast intraband relaxation in the conduction band on sub-ps time scale^36^, and subsequently relaxes back to the valence band to recombine with the hole (that is, exciton recombination). In contrast, in graded CQDs, both exciton recombination and charge transfer to a layer of larger-diameter CQDs can occur (Fig. 3a).

We carried out transient absorption measurements on graded CQD heterostructure films as well as on ungraded, single-sized CQD films. For the graded CQDs, we used three different sizes of CQDs with excitonic peaks at 950, 1,250 and 1,520 nm, while we used CQDs with excitonic peak at 950 nm for the single-sized CQDs. In the time-dependent transient absorption spectra shown in Fig. 3a, a positive feature is observed at wavelengths <950 nm for both single-sized CQDs and graded CQDs. This positive signal arises from bleaching of the lowest exciton transition in the smallest CQDs. The transient absorption signal of the graded CQDs shows an extra positive feature near 1,250 nm, which corresponds to the bleach of the exciton transition in larger CQDs. This bleach feature at 1,250 nm arises from excitons that were generated in high-lying excited states of 1250, nm bandgap CQDs and that rapidly relax to the lowest exciton states at 1,250 nm via intraband relaxation.

To examine the dynamics of electrons in the graded and single-sized CQDs, we compared the slices of the transient absorption signals at 950 nm as shown in Fig. 3b. The transient absorption signal of the single-sized CQDs decays with a single time constant of about 95 ps. The transient absorption signal of the graded CQDs exhibits an initial ∼8.4 ps exponential decay which we attribute to charge transfer between the layers containing different sizes of CQDs. Graded PbS CQD heterostructure films produced via conventional layer-by-layer assembly have been seen to exhibit a similar feature^37^. From this analysis we can estimate the charge transfer time (*τ*_CT_) using the following relation^38^


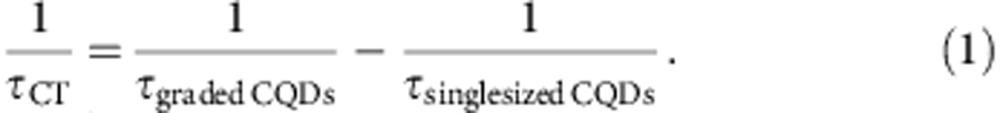


For the graded CQDs, *τ*_CT_ was determined to be 9.2 ps.

Although we observed a signature of charge transfer between CQDs of different sizes, it remained to be proven that this transfer is characteristic of graded CQD heterostructure, that is, the transfer from a layer of small CQDs to the one of larger CQDs. To resolve this issue, we also carried out transient absorption measurement on CQD films consisting of a random mixture of CQDs with the same mixing ratio that had been used in the graded CQD film. The transient absorption signal of the randomly mixed CQD films (Fig. 3b, Supplementary Fig. 10) exhibits more rapid decay (∼7.3 ps time constant) than that of the graded CQDs, giving a charge transfer time of ∼7.9 ps according to Eq. (1). The slightly faster charge transfer time in randomly mixed CQDs may be ascribed to the three-dimensional network of interfaces in the random case compared with the one-dimensional gradient case. Thus, the charge transfer in the graded CQDs is clearly distinguished from that in random CQDs. Of course, as explained in motivating this work, the graded case is necessary in achieving the intended directional transport.

To elucidate directional transfer in the graded films further, we investigated photoluminescence spectra for illumination from either the small-bandgap side or the large-gap side, with collection always on the small-gap side. We found that irrespective of whether illumination was from the small- or large-bandgap side, the emission spectra were dominated by small-bandgap emission (Fig. 3c,d). This confirms that when the large-gap side is illuminated, the photocarriers are efficiently conveyed to the small-gap material via the built-in gradient^37^,^39^ (Fig. 3c, top).

To verify this interpretation, we modelled this effect, calculating generation and recombination plots for the graded structures and using the electronic materials parameters previously reported for the CQD films used (Supplementary Table 2). In the model, just as in the experiments, recombination in the graded device occurs in the small-bandgap layers even for the case of generation is in the large-bandgap layers (Fig. 3c, bottom).

We further prove the charge carriers flow towards the potential energy minimum in the graded film using time-resolved photoluminescence (see Supplementary Fig. 11). A delayed onset of photoluminescence from the acceptor layers (that is, the smallest-bandgap) in graded film compared with the single-sized film account for carrier transfer of electronic excitations from the small dots to the large dots.

### Photodiode characteristics

We sought to identify CQD device categories that would uniquely benefit from the quantum funnel concept. Specifically, since quantum size tuning in PbS CQDs occurs predominantly in the conduction band, the graded heterostructure should propel minority electrons towards their collection electrode. This graded base should increase bandwidth in a photodetector and help fill factor in a solar cell.

**Figure 4:**
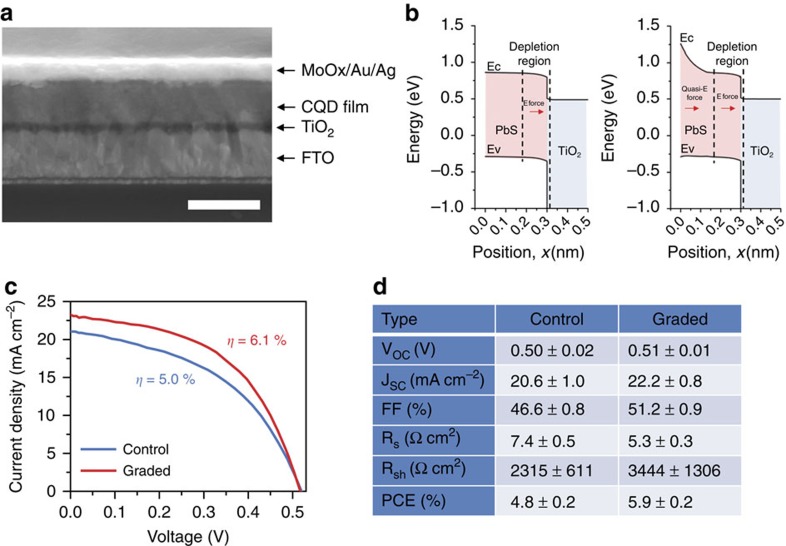
Photovoltaic cell performance of quantum-funnel CQD photodiodes. (**a**) A cross-sectional SEM image of a representative device. Scale bar, 500 nm. (**b**) Simulated spatial band diagrams of the ungraded control (left) and graded (right) devices operating at the maximum power point under AM 1.5 solar illumination. Band-bending near the rear of the device introduces a quasi-electric field capable of aiding carrier collection for improved performance. (**c**) Experimentally measured current–voltage characteristics under AM 1.5 simulated solar illumination for optimized devices employing ungraded control (blue) and graded (red) CQD devices. (**d**) Photovoltaic figures of merit of the devices cell under 100 mW cm^−2^ AM 1.5 illumination. *R*_s_ and *R*_sh_ are the series resistance and shunt resistance, respectively. Statistics for the devices are based on a total of >10 devices prepared on separate substrates.

We therefore fabricated graded-base photodiodes (Fig. 4a) using C3. We fabricated devices employing a CQD film (∼300 nm) whose grading comprised CQDs with excitonic peaks at 950, 850 and 750 nm. Because the lowest-bandgap dots deposit first out of solution, electrons are guided downward to the electron-extracting TiO_2_ electrode. The bandgap difference of 0.3 eV, principally felt in the conduction band, extends in graded manner over a 300-nm thick active region, adds a 10 kV cm^−1^ further built-in electric field due to the graded heterostructure (Fig. 4b, ungraded control versus graded device). Minimal size-effect tuning in the valence band ensures that majority hole transit to the top contact is not substantially impeded.

The current–voltage characteristics under AM 1.5 illumination (Fig. 4c) reveals improved short-circuit current density (*J*_SC_) and fill factor for the graded devices. The fill factor advantage is explained as follows: normally, as the control device moves from short-circuit conditions towards the maximum power *V*_MPP_, corresponding to a move from reverse bias in the direction of forward bias, the depletion region shrinks, and the quasi-neutral region expands from the back of the cell. The graded device retains the benefit of the built-in conduction band grading even as this happens, allowing it to retain operating current as it approaches the maximum power point. This effect is seen in the improved diode shunt resistance, which goes from 2,300±600 Ω cm^2^ (control) to 3,400±1,300 Ω cm^2^ (graded), driving the improvement in fill factor from 47±1 to 51±1%. The improvements in *J*_SC_ and fill factor combined produce an improved power conversion efficiency (PCE) from 4.8±0.2 to 5.9±0.2%. The best graded device showed a PCE of 6.1%, which is by a wide margin^30^ the highest PCE for a single-step-fabricated CQD active layer. To evaluate the homogeneity of our data, we analysed spatial variations of our C3-created film in different locations. The variation in average values of the device performance over the area is <5% (Supplementary Fig. 12).

As noted above, the prospective advantage in photodetector performance is expected to manifest in an increased speed of response. While this can be achieved in ungraded devices via a higher applied bias, this approach comes at the cost of higher dark current, which degrades signal-to-noise ratio and also mandates careful background subtraction. The quantum funnel photodiode design favours speed while retaining operation in photovoltaic-mode, thus enabling the sensitivity-bandwidth product to be maximized. Photovoltaic-mode light detection also eliminates electrical power consumption by the detector.

**Figure 5:**
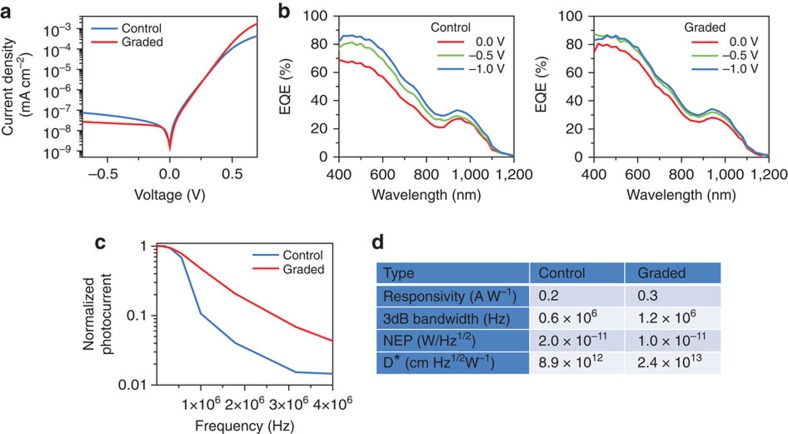
Photodetector performance of quantum-funnel CQD photodiodes. (**a**) Current–voltage characteristics in the dark for the control and graded CQD devices. The graded structure shows a significant reduction of the reverse saturation current density. (**b**) Spectral EQE for the control (left) and graded (right) CQD devices under varying reverse biases. Under larger reverse bias (−1 V), photogenerated charge collection is dominated by the external applied electric field and the devices have similar EQE profiles. However, compared with the control, the graded device is relatively insensitive to bias as a result of the conduction band funnel and shows a clear enhancement at zero bias. (**c**) Semilog 3 dB bandwidth plots for the control and graded CQD devices at zero bias. The 3 dB roll-off point increases from 0.6 to 1.2 MHz from the control to the graded device. (**d**) A summary of photodetector figures of merit for the control and graded devices at zero bias under 100 mW cm^−2^ of incident 640 nm light.

The dependence of external quantum efficiency (EQE) spectra (Fig. 5a) on electrical bias reveals the charge-collection dynamics of the devices. The ungraded device, with its low-collection-efficiency quasi-neutral region, benefits in its EQE spectrum from the application of a significant, depletion-region-expanding, reverse-bias. In contrast, the graded device is essentially bias invariant in its collection, maintaining high collection efficiency even down to zero bias—consistent with its super short-circuit current density *J*_SC_.

More striking still is the improvement in the temporal response of the graded device. It shows fully a doubling in speed of response at zero bias, its 3 dB bandwidth increasing from 0.6 to 1.2 MHz (Fig. 5b). The electron transit time is significantly decreased thanks to the appreciable additional driving force provided by the funnel acting primarily in the conduction band.

To determine the sensitivity of our quantum-funnel photodetector, we measured the light level required to obtain a signal-to-noise ratio of unity. This revealed a noise-equivalent power (NEP) of 2.0 × 10^−11^ W Hz^−1/2^ for the control and of 1.0 × 10^−11^ W Hz^−1/2^ for the graded device (Fig. 5d). The lower limit of detection in the graded device is also enhanced by its low dark current (Fig. 5c), related to its increased shunt resistance. The quantum-funnel device provides a normalized detectivity (D*) of ∼2.4 × 10^13^jones. This is the highest value for a CQD photodetector^40^,^41^.

## Discussion

In nanoparticle assembly, many external forces influence the particles and their interactions. The vectorial sum of these forces determines the direction and strength of the movement of the particles in the assembly kinetics.

In this work, we use external centrifugal forces to drive nanoparticle assembly kinetics in the vertical direction. They promoted an increase in the particle flux settling to the substrate under the centrifugal field, and increased the rate of particle–substrate interaction to advance the assembly process.

We took the view that if an optoelectronic-quality CQD solid can be directly fabricated via C3, a number of advantages would be provided. Centrifugal casting shown here enables ease of fabrication of a uniform CQD film over large areas with low materials waste via directed assembly using centrifugal acceleration, and achieves this using a single processing step. This approach has the potential to enable the fabrication of gradient nanoparticle films using a single step. Further, C3 method can produce a very well-defined gradient in CQD size within a film: in the presence of polydispersity within a single batch of dots, conventional deposition methods such as dip coating will not remedy this polydispersity within the film formed, whereas the gradient approach will sort systematically according to size.

The resultant quantum-size-gradient devices are of interest not only in fast photodiodes, but also in graded-based heterojunction bipolar transistors, back-surface fields for reduced recombination in solar cells, and continuously, spectrally, tunable light-emitting devices. This work highlights the benefits of size/bandgap-gradient devices, and introduces a single-step means of fabricating them using a manufacturing method that can now be applied to novel, optoelectronics-ready, nanoparticle colloids.

## Methods

### Colloidal quantum dot synthesis

The PbS CQDs were synthesized based on a previously published method that incorporated an in-synthesis solution-phase metal halide (CdCl_2_) treatment step^42^,^43^. During synthesis, the dots were treated with 1.0 ml of CdCl_2_ and tetradecylphosphonic acid in oleylamine at a 13.6:1 molar ratio. This solution was incorporated into the reaction after adding the sulfur precursor during the cooling process when the temperature had reached 60 °C. The CQDs were precipitated with acetone and then centrifuged as the temperature reached 30–35 °C. The CQD material was then dried using a nitrogen stream, redispersed in toluene and centrifuged after addition of equal volumes of acetone and methanol. The precipitated CQD material was dried under vacuum to remove any residual solvent then redispersed in anhydrous toluene. Three additional redispersion/precipitation cycles were carried out in methanol before final drying and dispersion in octane to produce a solution with concentration of 50 mg ml^−1^ in octane.

### Ligand exchange

The ligand exchange process was carried out in air atmosphere. For a typical ligand exchange with MPA molecules, 3 ml of OA–PbS CQD solution (∼2.5 mg ml^−1^ in octane) was mixed with 3 ml of dilute solution of MPA (∼60 mg ml^−1^ in DMSO). The mixture was vortexed vigorously for 2 min, leading to a complete phase transfer of PbS CQDs from octane to the DMSO phase. The phase transfer was easily monitored by the colour change of solvent phases. Then the DMSO phase was separated out followed by triple washing with octane to remove any remaining non-polar organic species. Finally, the exchanged CQD solution was diluted to a concentration of ∼0.75 mg ml^−1^ by adding more DMSO solvent to it before film fabrication.

### Film fabrication via centrifugal casting

To form the CQD film, the MPA-exchanged PbS CQD solution (typically 20 ml with a concentration of ∼0.75 mg ml^−1^ in DMSO) was added in 30-ml centrifuge tube, together with the substrate placed inside a centrifuge tube. The tilt angle (∼30°) and rotation speed (∼15,000 *g*) were optimized to allow for the uniform casting from the CQD solution under centrifugation. Centrifugal sedimentation rate of CQDs are allowed to control with adding small amounts of marginal solvents into the CQD ink. Centrifugation time was determined empirically by waiting for the CQD solution to become clear and colourless to the eye (typically <1 h). This supernatant was then discarded and the substrate gently removed from the tube and rinsed with DMSO to ensure removal of residual organics before final drying the film via spinning at room temperature in air. Film thickness was controlled by varying the concentration of CQD solution.

### Transient absorption spectroscopy measurement

Transient absorption spectra were measured with femtosecond laser pulses using a visible pump–broadband probe scheme. The output pulses at 800 nm wavelength from a Ti:sapphire amplified laser (Coherent Legend Elite) were split into pump and probe arms. On the pump arm, the 800 nm laser pulses were converted into the pump pulses of 540 nm wavelength and 30-nm bandwidth using a home-built, all-reflective-optic noncollinear optical parametric amplifier. The pump pulses were sent through a pair of fused-silica prisms to precompensate for the dispersion obtained from transmissive optics and compressed to near-transform-limited pulses at the sample position. The pulse energy of the pump pulses was adjusted to ∼60 nJ at the sample position to suppress the exciton–exciton annihilation process. On the probe arm, the 800 nm laser pulses were sent into a *c*-cut sapphire window of 3-mm thickness and converted into white light continuum spanning from visible to near-infrared wavelengths by self-phase modulation. The near-infrared portion (850–1350, nm) of the white light continuum was used as broadband probe pulses without further compensation of the dispersion. The probe pulses were time-delayed with respect to the pump pulses using a motorized translation stage (Newport, M-ILS150HA). A weak portion of the probe pulses were split and used as a reference to calculate the fractional transient absorption. The spectra of transient absorption signal and the reference were detected by a spectrometer (Andor, SR303) equipped with an InGaAs CCD (Andor, DU491A-1.7). In all the measurements, the polarization of the pump pulses was set to be 54.7° relative to the probe polarization. The time resolution of the transient absorption measurement was ∼50 fs according to the cross correlation function of the pump and the probe pulses measured at the sample position using a coherent spike from pure solvent (ethanol). For each sample, the transient absorption spectra were measured in the time range up to 100 ps.

### Photodiode device fabrication

Devices were fabricated on TiO_2_ n-type electrodes. TiO_2_ thin films of ∼50 nm were deposited on FTO-coated glass (TEC15, Hartford Glass) using d.c. magnetron sputtering. The substrates were then treated with a 120 mM TiCl_4_ solution and baked at 70 °C for 30 min in an oven. The substrates were then dried and annealed for 30 min at 520 °C on a hotplate in air. Then, CQD film deposition was performed onto the electrodes via centrifugal casting. Metal contacts (7.5 nm of MoO_3_, 50 nm of Au and 120 nm of Ag) were deposited with an Angstrom Engineering Åmod deposition system inside in an Innovative Technology glovebox.

### AM 1.5 photovoltaic device characterization

Devices were illuminated through a 0.049-cm^2^ aperture with a Solar Light XPS 200 solar simulator at an irradiance of 100 mW cm^−2^. The power was measured using a calibrated Melles-Griot power meter. A Keithley 2400 source meter was used to apply a bias and to measure current. A calibrated reference solar cell from Newport was used to measure and account for the spectral mismatch between the solar simulator and the true AM 1.5G spectrum. The estimated mismatch was 5%, and all current values were multiplied by 0.95 to account for this. The estimated uncertainty of the AM 1.5G measurements is±7%.

### External quantum efficiency measurements

Monochromatic light from a filtered 400-W xenon arc lamp source on top of a constant one-sunlight bias was incident on the device. The monochromatic light was mechanically chopped at 220 kHz. Calibrated Newport 818-UV and 818-IR power meters were used to measure the monochromatic light power. Current was measured with a SRS lock-in amplifier at short-circuit conditions. The propagated root-mean-squared error of all equipment used in these measurements, as well as device active area, was estimated to be±3%.

### Bandwidth measurements

Photodiode devices were illuminated by 640 nm laser diode modulated by an Agilent 33220A 20 MHz function generator. Photocurrent measurements were conducted using a 25-V resistive load as a transimpedance stage and a Tektronix TDS 5104 digital oscilloscope. High illumination intensities (100 mW cm^−2^) were used to provide suitable signal-to-noise ratios for measurements. All measurements were performed in the dark, shielded enclosure at room temperature in an N_2_ atmosphere.

### Noise measurements

Noise current in the photodetectors was measured in the dark, at zero bias, using a Stanford Research SR830 lock-in Amplifier in current measurement mode. The reported dark noise current, normalized to the measurement bandwidth, divided by the responsivity under the same measurement conditions, yielded the NEP. The normalized detectivity D* was obtained as the reciprocal of NEP normalized by the square root of the optically active area of the device and the bandwidth:


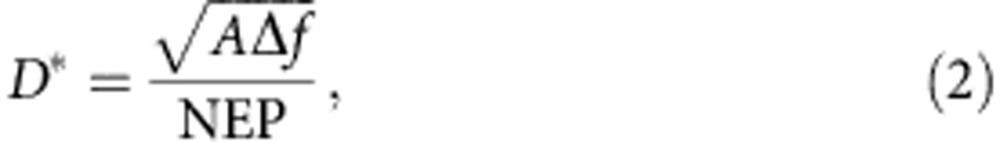


where A is the area of the photosensitive region and Δ*f* is the frequency bandwidth of the device. All measurements were performed in a dark, shielded enclosure at room temperature in an N_2_ atmosphere.

## Additional information

**How to cite this article:** Kim, J. Y. *et al.* Single-step fabrication of quantum funnels via centrifugal colloidal casting of nanoparticle films. *Nat. Commun.* 6:7772 doi: 10.1038/ncomms8772 (2015).

## Supplementary Material

Supplementary Information Supplementary Figures 1-12, Supplementary Tables 1-2, Supplementary Note 1 and Supplementary References
