# Supplementary material for: Single-step fabrication of quantum funnels via centrifugal colloidal casting of nanoparticle films
Source: Nat Commun. 2015 Jul 13;6:7772. doi: 10.1038/ncomms8772 (PMC4510961; doi:10.1038/ncomms8772)
Supplement: Supplementary Information — Supplementary Figures 1-12, Supplementary Tables 1-2, Supplementary Note 1 and Supplementary References [file ncomms8772-s1.pdf]

Supplementary Figures

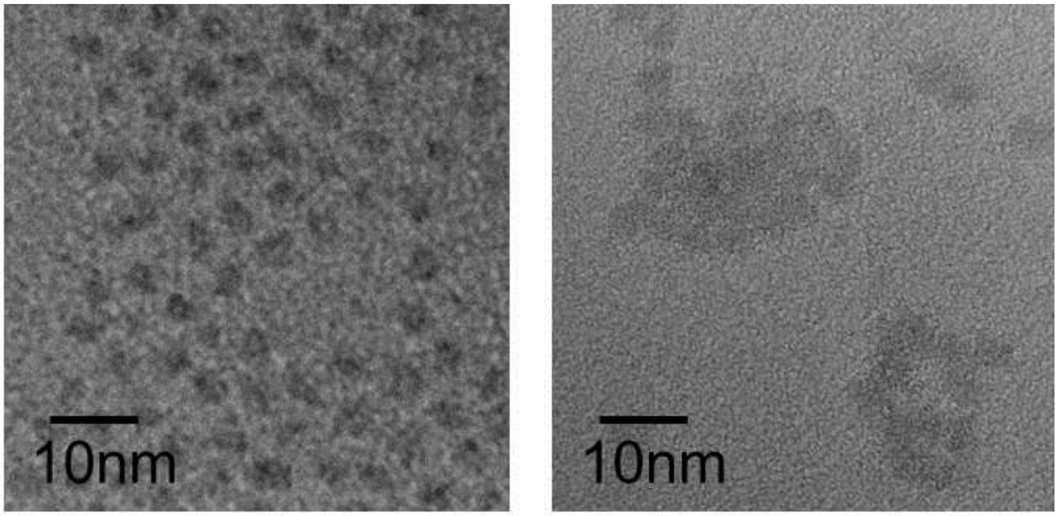

Supplementary Figure 1. TEM images of PbS CQDs before (left) and after (right) ligand exchange with MPA.

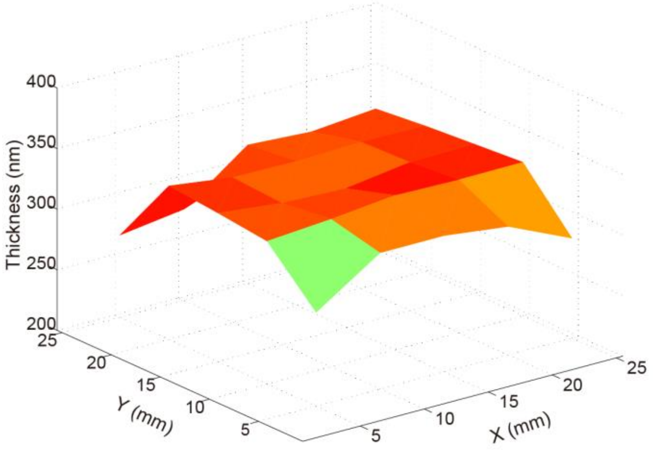

Supplementary Figure 2. Spatial variation of thicknesses with position on a CQD film via C3 processing.

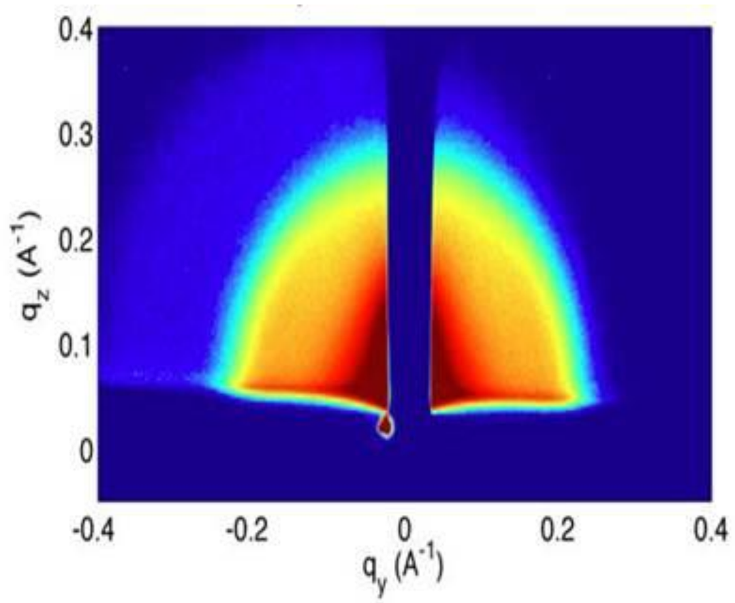

Supplementary Figure 3. GISAXS intensity pattern of PbS CQD film made by centrifugal casting. The axes represent the scattering wave vectors in the plane of the substrate, $q_{x,y}$ (scattering in the plane of the substrate) in the z direction, q_z (scattering normal to the plane of the substrate), respectively. The color scale represents the log of the scattering intensity as recorded by the CCD detector. Blue represents lower intensity and red represents higher intensity, with each spectrum normalized to show the full dynamic range of each data set.

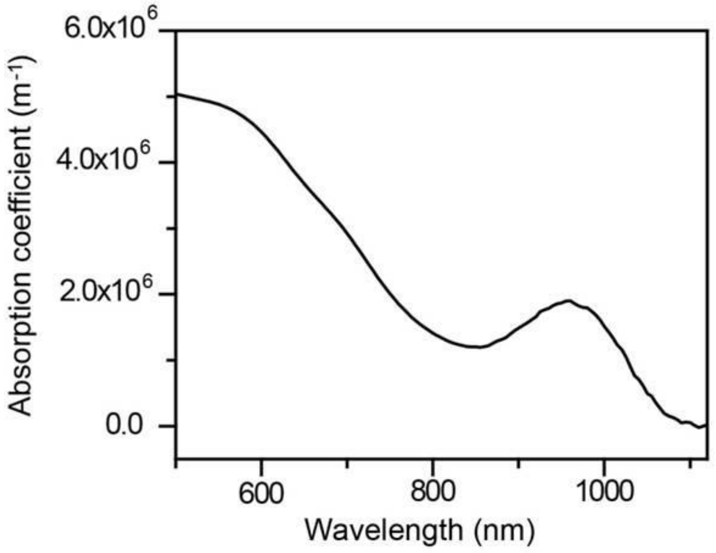

Supplementary Figure 4. Absorption spectrum for MPA-exchanged PbS CQD film made by centrifugal casting.

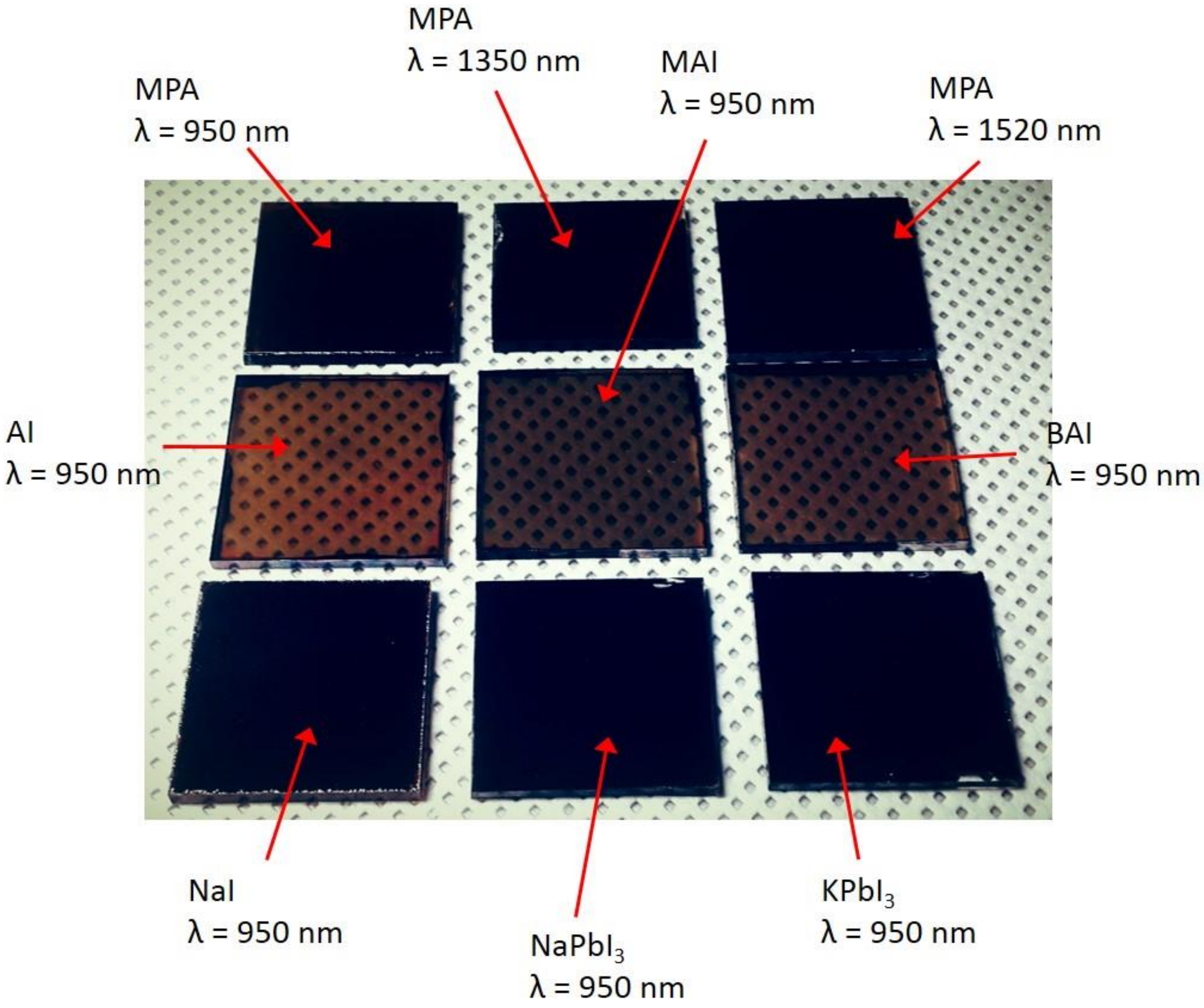

Supplementary Figure 5. Photograph showing C3-created CQD films; MPA-PbS CQD films with excitonic peak at 950, 1350 and 1520 nm (top) and PbS CQD films capped with various organic (middle) and inorganic ligands (bottom): NH_4I (AI), CH_3NH_3I (MAI), $C_4H_9NH_3I$ (BAI), NaI, $NaPbI_3$ and $KPbI_3$.

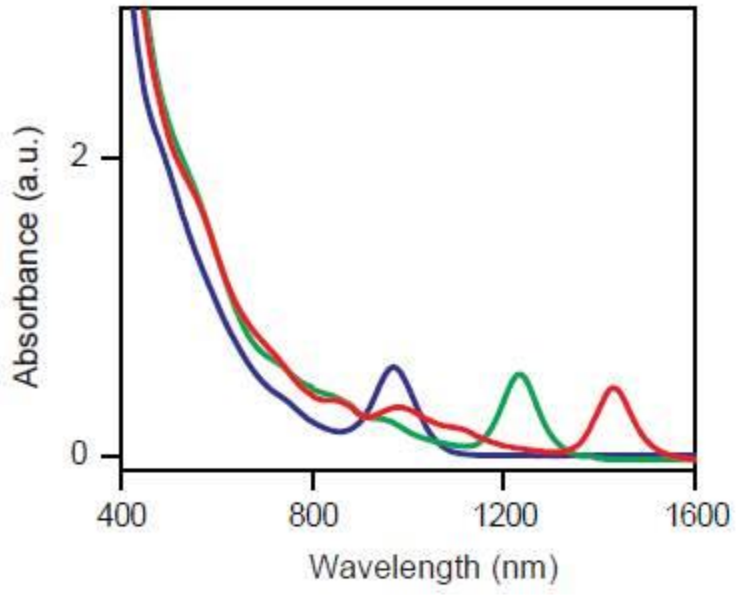

Supplementary Figure 6. Absorption spectra for MPA-exchanged PbS CQD solution with excitonic peaks of 950 (blue), 1250 (green) and 1520 nm (red).

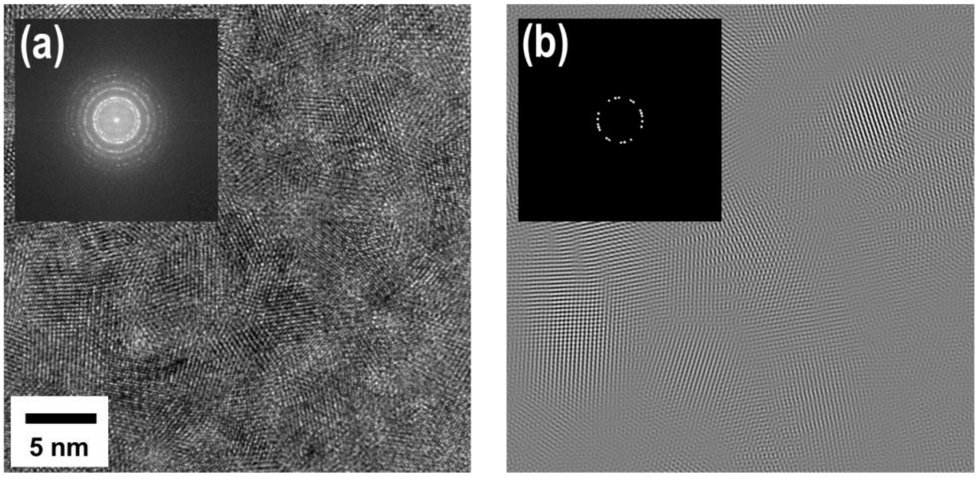

Supplementary Figure 7. (a) High-resolution TEM (HRTEM) image of MPA-PbS CQDs. (Inset) fast Fourier transform (FFT) image and (b) Inverse FFTed image after Bragg filtering of the original HRTEM image with inset of FFT image.

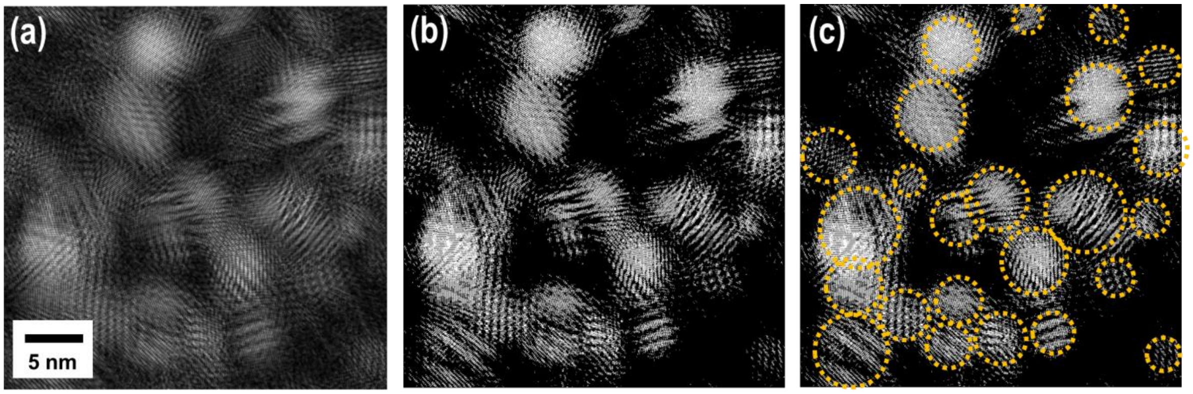

Supplementary Figure 8. (a) Mean-square filtered image, (b) high-pass filtered image after the mean-square filtering, and (c) size-configured small grains encircled with yellow dot lines in the high-pass filtered image using algorithm for cluster configuration analysis.

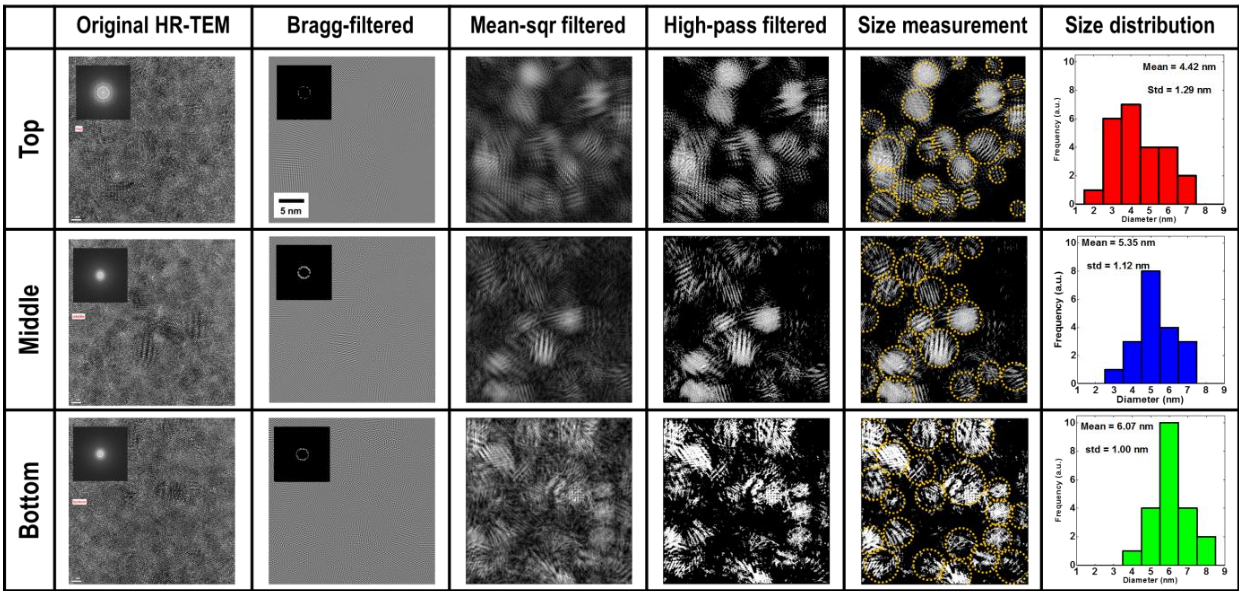

Supplementary Figure 9. Summary showing the image processing steps for the particle size measurement and the histograms of size analysis results obtained at the different positions in the graded PbS CQD structure (top, middle and bottom in Fig. 2c).

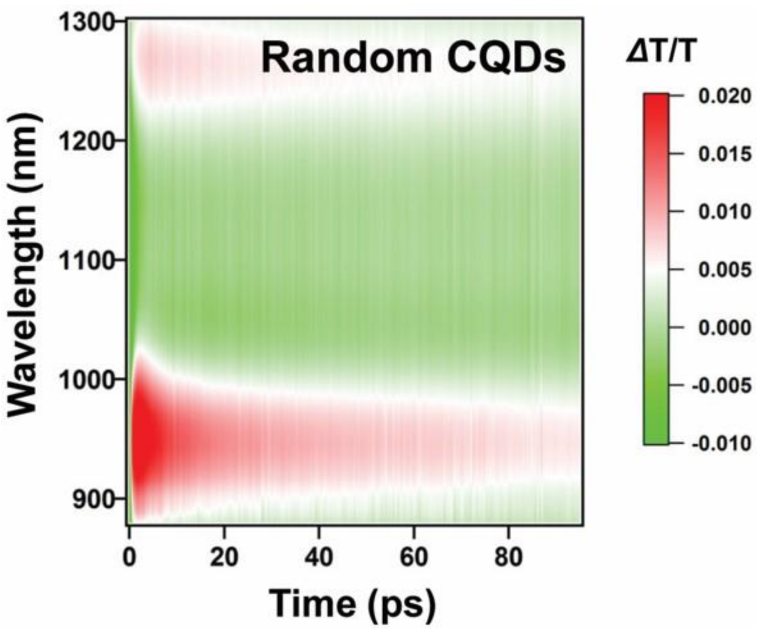

Supplementary Figure 10. Time evolution of TA spectra for randomly mixed CQDs.

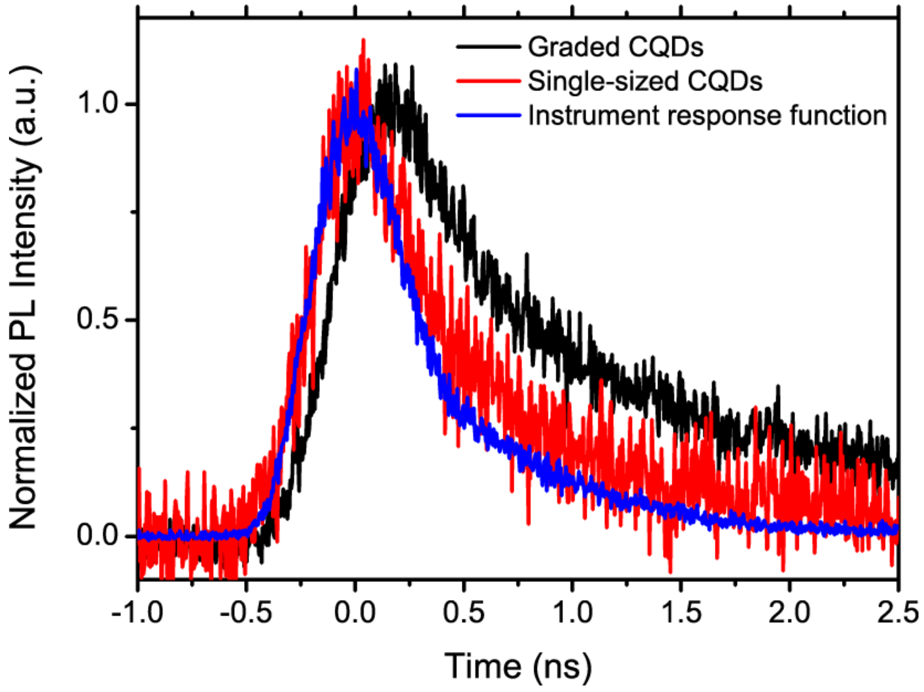

Supplementary Figure 11. Time resolved photoluminescence spectra measured at 1520 nm CQDs in single-sized and graded CQD solids.

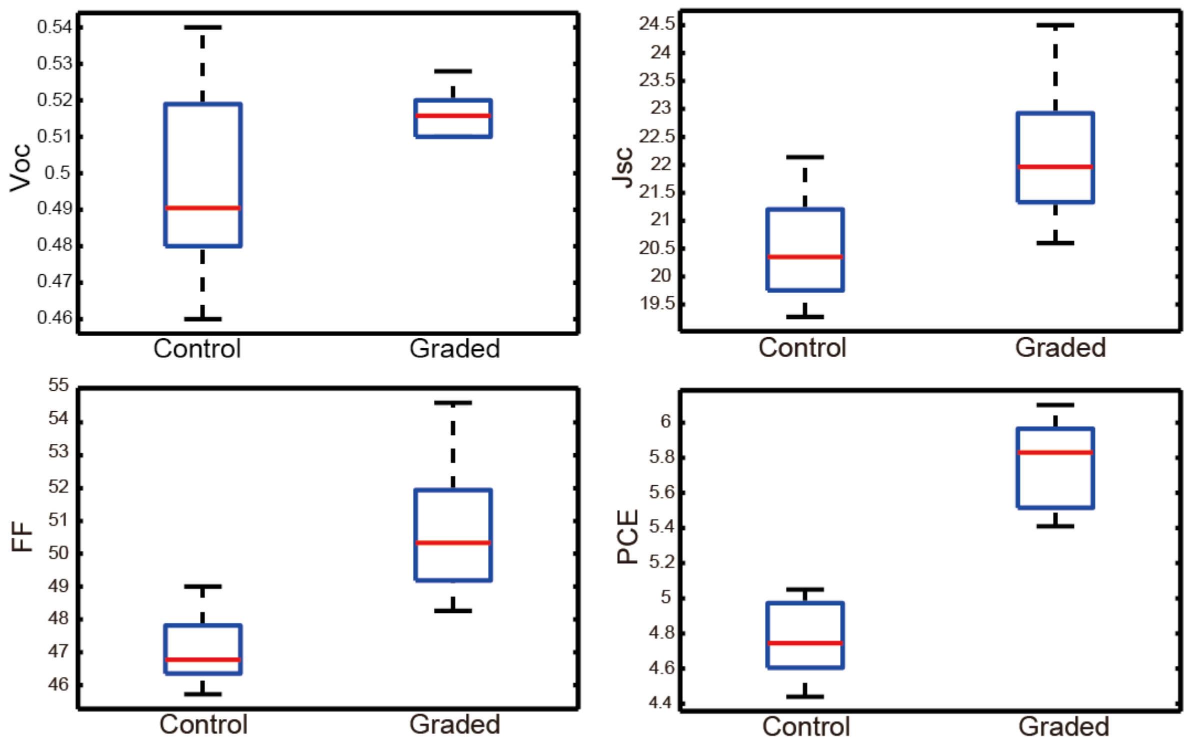

Supplementary Figure 12. Spatial variation of PV device parameters of the ungraded control and graded devices under AM 1.5 solar illumination. For each box plot, the horizontal line represents the median of the data set, and the box shows the interquartile range. The whiskers represent the standard deviation.

Supplementary Tables

Supplementary Table 1. A summarized computational procedure to quantitatively measure the cluster size based on the algorithm for cluster configuration analysis (ACCA).

**Algorithm for Cluster Configuration Analysis (ACCA) for the size measurement of particles in the image**
Step 1. Set initial coordinates (considering self-exclusion effects) of pixels in the image
Step 2. Find a list of nearest neighbors (nns) for each of the pixels within the cutoff distance
Step 3. Find monomers (pixels with no nns)
Step 4. For the remaining pixels (not monomers) accompanied by respective nns<br>Step 4.1. Find a list of nns of the 1st pixels (≡ $\{NN_1\}$), and make a set for pixels indices involved in the 1st cluster (≡ $\{CLUS_1\}$) including the 1st pixel itself and its nns<br>Step 4.2. For the ith nn pixel of the 1st particle (n_{1i}), find a list of nns of the 1th nn pixels ($\{NN_{n1i}\}$) and update $\{CLUS_1\}$ such that $\{CLUS_{1\_new}\} = \{CLUS_{1\_old}\} \cup \{NN_{n1i}\}$<br>Step 4.3. Repeat Step 4.2 until $\{CLUS_{1\_new}\} = \{CLUS_{1\_old}\}$<br>Step 4.4. If $\{CLUS_{1\_new}\} = \{CLUS_{1\_old}\}$, set $\{CLUS_1\} = \{CLUS_{1\_new}\}$ and exclude pixel indices in $\{CLUS_1\}$ from entire pixel' indices and repeat Step 4 to construct $\{CLUS_2\}$ by repeating Steps 4.1-4.3.<br>Step 4.5. Repeat Step 4 until there are no pixels to be included in clusters.
**End of Algorithm**

Supplementary Table 2. Summary of the material properties used in the optoelectronic simulation used in the study. SCAPS 3.0.01 was used for the optoelectronic simulations. The film structure was simulated from Fig. 3b of the main text. The following table summarizes the material properties used:

Material	Thickness (μm)	Doping (cm^{-3})	$\mu_{e,h}$ (cm^2/Vs)	Bandgap* (eV)	Electron affinity (eV)	α (cm^{-1})
PbS	Variable	$3.5e^{16}$	$1.3e^{-2}$	1.3	3.95	From Spectrum
TiO_2	0.5	$5e^{18}$	$5e^{-2}$	3.2	4.08	From Spectrum

*As defined by absorption: excitonic peak position

Supplementary Note

Supplementary Note 1: Analysis of nanoparticle size using image analysis methods based on Fourier filtering techniques.

Our numerical routine for the image analysis includes the following:

Fast Fourier transform (FFT). The PbS CQDs in the film have crystalline structure, and therefore, they are observed as grains with lattice-structures in the HRTEM image (refer to Supplementary Fig. 6). This indicates the existence of the characteristic periodicity corresponding to the lattice constant, and this periodicity can be directly observed as ring or dot patterns in the FFT image as shown in the inset of Supplementary Fig. 6. From the FFT image, the concentric ring patterns are responsible for the small grain size in which lattice constants of the grains are equivalent but their orientations are different.

Bragg filtering. Although FFT provides quantitative information on the lattice constant of the small grains of the CQDs, can little be extracted from the FFT images. In order to obtain the quantitative information on the size of the small grains, we can apply the Bragg filtering which scans Fourier transformed amplitudes along the circumstances of the concentric rings obtained in the FFT image. As is in the context of the theoretical approach suggested by Hÿtch[1,2], overlapping the amplitudes extracted from the Bragg filtering enables constructing a map which provides selectively intensified grain images from the original HRTEM image. The amplitude, $A_{\mathbf{k}}(\mathbf{r})$, can be obtained using Fourier summation of the raw HRTEM image signals ($I(\mathbf{r})$) as

$$I(\mathbf{r}) = \sum_{\mathbf{k}} A_{\mathbf{k}}(\mathbf{r}) \exp\left(iP_{\mathbf{k}}(\mathbf{r})\right) \exp\left(i2\pi \mathbf{k}\cdot\mathbf{r}\right), \quad (1)$$

where $\mathbf{k}$ denotes the reciprocal wave vector and $P_{\mathbf{k}}(\mathbf{r})$ is the phase at the position $\mathbf{r}$. For the Bragg filtering, a circular window numerically scans a circular path along each concentric ring in the FFT image, and the scanning resolution was set as $1°$ in order to reduce numerical error. The scanning window size was set to sufficiently cover the Bragg spots located in the concentric rings, but to be small not to impinge two or more concentric rings simultaneously. With the

overlapped Bragg filtered amplitudes, we can construct an amplitude map in the reciprocal space, and obtain an inverse FFTed image, subsequently as shown in Supplementary Fig. 6b.

Mean-square filtering (Bilateral range filtering). Bragg filtering provides numerically reconstructed grains which are different from background noise. However, it is still hard to delineate the CQDs, since the grains signals are not intensified. In order to intensify the filtered amplitudes of the lattice-structures in the small grains, we applied mean-square filter (or bilateral black-and-white filter) by which a square window scans and bilaterally filters the inverse FFTed image with shift-invariant Gaussian filters with the reference signal value set as the mean of the signals obtained from the inverse FFTed image[3]. We examine several sets of window sizes and bilateral patterns, and chose a combination to obtain the best filtered image as shown in Supplementary Fig. 7a. Comparing to the Bragg-filtered images, we can find that the mean-square filtered image can effectively capture the small grains composed of lattice structures with highly intensified and distinguishable shapes.

High-pass filtering. Finally, we applied a high-pass filter by which the mean-square filtered image is converted into black-and-white image as provided in Supplementary Fig. 7b. Using the high-pass filtering, we can obtain a matrix for the filtered image and the filtered signals can be converted into coordinates of pixels.

Size measurement. Using the coordinates, we can apply an algorithm to measure the size of the small grains (as algorithm for cluster configuration analysis (ACCA)[4]. The detailed procedure to run the ACCA is provided in Supplementary Table 1. By using the ACCA, we can quantitatively distinguish the particle from other particles. For the application of the ACCA, one needs to set the threshold distance to distinguish the nearest neighbors and other pixels, and we set the threshold as 1.1 times of the lattice constant observed in the FFT image. And the size was calculated with the definition of the radius of gyration, R_g , as

$$R_g = \left(\sum_{\mathbf{r}} \left| \mathbf{r} - \mathbf{r_0} \right|^2 \right)^{1/2}, \tag{2}$$

where $\mathbf{r}_0$ is the position of the pixel which has the minimum total inter-distance among other pixels in the same grain at the position $\mathbf{r}$. This position works as center of mass to configure each of the CQDs in the image. In Supplementary Fig. 7c, we provided a result obtained from numerical analysis of the size measurement for the small grains from the high-pass-filtered image. Each of the circles is drawn around the respective center of mass positions with the respective radius measured by the ACCA.

Supplementary References

1. Hÿtch, M. J. Analysis of variations in structure from high resolution electron microscope images by combining real space and Fourier space information. *Microsc. Microanal. Microstruct.* **8**, 41–57 (1997).

2. Seyring, M., Song, X., Chuvilin, A., Kaiser, U. & Rettenmayr, M. Characterization of grain structure in nanocrystalline gadolinium by high-resolution transmission electron microscopy. *J. Mater. Res.* **24**, 342–346 (2009).

3. Tomasi, C. & Manduchi, R. Bilateral filtering for gray and color images. *Proc. IEEE Int'l Conf. Comp. Vis.* 839–846 (1998).

4. Kwon, S. J. Phase-equilibrium-mediated assembly of colloidal nanoparticles. PhD dissertation, Massachusetts Institute of Technology (2013).
